# Hepatitis C Virus Core Protein Down-Regulates Expression of Src-Homology 2 Domain Containing Protein Tyrosine Phosphatase by Modulating Promoter DNA Methylation

**DOI:** 10.3390/v13122514

**Published:** 2021-12-15

**Authors:** Priya Devi, Seisuke Ota, Tanel Punga, Anders Bergqvist

**Affiliations:** 1 Department of Medical Sciences, Uppsala University, SE 751 85 Uppsala, Sweden; Priya.Devi@medsci.uu.se; 2 Department of Internal Medicine, The Himeji St. Mary’s Hospital, Himeji 670-0801, Japan; seisukeota@yahoo.co.jp; 3 Department of Medical Biochemistry and Microbiology, Uppsala University, SE 751 23 Uppsala, Sweden; Tanel.Punga@imbim.uu.se; 4 Clinical Microbiology and Hospital Infection Control, Uppsala University Hospital, SE 751 85 Uppsala, Sweden

**Keywords:** HCV, core protein, phosphotyrosine, SHP-1, CpG, methylation

## Abstract

Hepatitis C virus (HCV) is the major causative pathogen associated with liver cirrhosis and hepatocellular carcinoma. The main virion component, the core (C) protein, has been implicated in several aspects of HCV pathology including oncogenesis and immune subversion. Here we show that expression of the C protein induced specific tyrosine phosphorylation of the TCR-related signaling proteins ZAP-70, LAT and PLC-γ in the T cells. Stable expression of the C protein specifically reduced Src homology domain 2-containing protein tyrosine phosphatase 1 (SHP-1) mRNA and protein accumulation. Quantitative CpG methylation analysis revealed a distinct CpG methylation pattern at the SHP-1 gene promoter in the C protein expressing cells that included specific hypermethylation of the binding site for Sp1 transcription factor. Collectively, our results suggest that HCV may suppress immune responses and facilitate its own persistence by deregulating phosphotyrosine signaling via repressive epigenetic CpG modification at the SHP-1 promoter in the T cells.

## 1. Introduction

Hepatitis C virus (HCV) is one of the main causes of liver inflammation in humans. The virus can cause short-term acute infections which are self-limiting and relatively rare. More commonly, HCV causes long-term persistent infections, eventually leading to liver diseases, which may ultimately progress to liver (hepatocellular carcinoma, HCC) and non-liver (lymphoma) cancers [1,2,3]. HCV is mainly a liver-specific virus, but many studies have reported the extra-hepatic replication of virus in peripheral blood mononuclear cells (PBMCs) [4,5,6,7]. Notably, monocytes, macrophages, dendritic cells, T and B cells support the HCV infection in vitro and ex vivo, particularly when isolated from the immunocompromised patients [8]. The extra-hepatic proliferation could serve as a reservoir for persistent HCV infection, with a potential for virus reactivation [9]. Although the exact molecular mechanisms of HCV persistent infection have remained obscure, it has been proposed that the virus establishes the long-term infection by evading the host immune responses [10,11]. For example, activation of virus-specific cytotoxic T lymphocyte (CTL) is critical for the elimination of virus infected cells. Virus-specific CTL are significantly diminished in chronic HCV patients compared to patients infected with other persistent viruses, such as hepatitis B virus (HBV) and human immunodeficiency virus type 1 (HIV-1) [12,13].

HCV core (C) protein is the first protein to be synthesized by the virus, which after cleavage from the polyprotein by host proteases exhibits different cellular localization such as nucleus, endoplasmic reticulum, lipid droplet, lipid raft and mitochondria. The C protein interacts with different cellular proteins and is involved in processes like cell cycle, apoptosis, autophagy and tumorigenesis [14]. The C protein also interacts with different components of the immune system to facilitate virus persistence [15,16,17]. Chronicity is associated with dysfunctional T cell responses and the C protein can interfere with T-cell signaling [18]. It has been previously shown that expression of HCV C gene product interferes with the Ca^2+^ signaling and disbalances the homeostasis in the biological system [19]. The Ca^2+^ efflux in turn upregulates the proximal signaling by inducing anergy-associated genes including soluble and receptor tyrosine phosphatases [20,21].

Protein tyrosine kinases (PTKs) and protein tyrosine phosphatases (PTPs) are the two key components of T cell signal transduction required for the effector T cell function. Various PTKs activate the signaling proteins by phosphorylating particular tyrosine residues while the PTPs terminate the activation by dephosphorylating the target proteins. Together, PTKs and PTPs maintain the feedback control loop by activating or inhibiting phosphotyrosine signaling [22]. The T cells express approximately 60 PTPs divided into transmembrane, receptor PTPs (RPTPs), and cytoplasmic, non-receptor PTPs (NRPTPs) [23]. The RPTPs such as CD45, CD148 and SHP-2 are relatively less expressed in T cells and mostly have a positive role in T cell receptor (TCR) signaling [24,25,26,27]. The Src-homology region 2 domain-containing tyrosine phosphatase (hereafter as SHP-1, but also known as PTPN6, SHP, SHPTP-1, HCP, PTP1C) [28,29]) is a NRPTP, which negatively regulates the TCR, cytokine and inflammatory signaling by dephosphorylating the receptor-associated kinases and other kinases [30,31]. The SHP-1 gene is present on chromosome 12 in humans and contains two different cell-specific promoters. In the hematopoietic cells SHP-1 is expressed from promoter 2 (P2), while low levels of SHP-1 expression occur from promoter 1 (P1) in epithelial cells [32].

The SHP-1 is also a tumor suppressor gene and reduced SHP-1 expression owing to the promoter hypermethylation has been reported in different blood-related cancers [33,34,35,36,37,38,39,40,41]. Promoter methylation is achieved by addition of a methyl group to cytosine in the CpG dinucleotide context by the DNA methyltransferases (DNMTs). There are different types of DNMTs in eukaryotic cells but DNMT1, DNMT3A and DNMT3B are the main methyl transferases that are actively involved in the CpG methylation. DNMT3A and DNMT3B are the de novo methyltransferases that establish CpG methylation during the early stages of development while DNMT1 is the maintenance enzyme and copies CpG methylation pattern on parental strand to the new strand during DNA replication [42]. A recent study has shown the deregulation of SHP-1 expression in HCC and a correlation of reduced SHP-1 expression with poor patient survival in hepatitis B virus (HBV) positive HCC [43].

In the present investigation, we examined the effects of HCV C protein on phosphotyrosine signaling in T cells. We demonstrate that expression of the HCV C protein induces a particular CpG methylation pattern at the SHP-1 gene promoter. This epigenetic modification correlates with reduced SHP-1 mRNA expression and general dysregulation of the phosphotyrosine signaling.

## 2. Materials and Methods

### 2.1. Chemicals and Antibodies

DNA methyltransferase inhibitor 5-Aza-2′-deoxycytidine (5-Aza) and puromycin were purchased from Sigma-Aldrich (Stockholm, Sweden). The mouse anti-CD3 antibody (HIT3a) was from BD Biosciences (Stockholm, Sweden). Antibodies to detect phosphorylated forms of PLC-γ1 (Tyr783), p-ZAP-70 (Tyr493) and p-LAT (Tyr171) were purchased from Cell Signaling Technology (Leiden, Netherlands). Antibodies to detect the PLC-γ1, ZAP-70, Lck, HCV C, CD45, SHP-1, SHP-2, actin and the pan-phosphotyrosine antibody (PY99) were purchased from Santa Cruz Biotechnology (Heidelberg, Germany). PE-conjugated SH-PTP1 (C-19, sc-287) and I_g_G, isotype control antibody for flow cytometry (FC) was purchased from Santa Cruz Biotechnology. Trypan blue stain (0.4%) was from Thermo Fisher Scientific (Uppsala, Sweden).

### 2.2. Cell Culture

The human Jurkat T cell (subclone E6-1) and its Lck kinase deficient derivative, J.Cam1, were obtained from American Tissue Type Collection. Three HCV C protein-expressing Jurkat T cell lines (JHC.d, JHC.g and JHC.h) contain DNA sequence from the infectious HCV H77 strain encoding the first 194 amino acid of the HCV polyprotein as described previously [17,18]. Single cell clones were selected on puromycin (0.2 µg/mL final concentration) and tested for the C protein expression by western blotting. Human monocytic THP-1 cell line was kindly provided by Dr. Göran Akusjärvi, Uppsala University, Uppsala, Sweden. The human embryonic kidney cell line HEK293TT expressing high levels of SV40 large T-antigen was kindly provided by Dr. Helena Faust [44]. The Jurkat, J.Cam1, JHC.d, JHC.g, JHC.h (collectively as JHC) and THP-1 cell lines were cultured in RPMI-1640 media (Sigma-Aldrich) supplemented with 10% fetal calf serum (FCS) and 1% penicillin-streptomycin mixture (Sigma-Aldrich) whereas 293TT cells were cultured in DMEM (high glucose) medium (Sigma-Aldrich) with 10% FCS and 1% penicillin-streptomycin mixture at 37 °C in a 5% CO_2_ humidified atmosphere.

### 2.3. Cell Lysis and Western Blot

Jurkat cells (5 × 10^6^) or its derivatives were stimulated by treating cells with an anti-CD3 antibody for 5 min at 37 °C. Cells were washed with PBS, pelleted and solubilized by resuspending and heating at 95 °C for 5 min in an SDS-PAGE sample buffer or lysed in NP-40 buffer (50 mM Tris-Cl, pH 7.5; 150 mM NaCl and 1% NP-40) for 20 min on ice. Proteins were resolved on 8, 10, or 12% SDS-PAGE and transferred to a 0.45 or 0.2 µm Amersham nitrocellulose membrane (GE Healthcare, Uppsala, Sweden) using a wet blotting apparatus. The primary antibodies were used at 1:2000 to 1:100 of final dilution. Proteins were detected with secondary horseradish peroxidase-linked (Dako A/S, Sundbyberg, Sweden) or fluorescent-labeled IRDye (LI-COR Biotechnology, Homburg Germany) secondary antibodies. The proteins were visualized with Pierce enhanced chemiluminescence (Thermo Fisher Scientific) or scanned using an Odyssey scanner (LI-COR).

### 2.4. RT-qPCR

About 1 × 10^6^ cells were harvested, washed in PBS and total RNA was isolated using RNeasy plus mini kit (Qiagen, Hilden, Germany). The cDNA synthesis and quantitation of SHP-1 and β-Actin mRNA were performed using 400 nM of each primer, 200 nM of probe and the QuantiTect Virus master mix (Qiagen) in PCR program for RT was 50 °C for 30 min, denaturation at 95 °C for 15 min, and 45 cycles of 95 °C for 15 s and 60 °C for 60 s. The primers and probes for RT-qPCR of SHP-1 mRNA were as follows: SHP-1-For, 5-GCTACAACATTCTCCCCTTTGAC-3; SHP-1-Rev; 5-GGGCCTAGCTGGTTCTTGATGTA-3; SHP-1-Pr, 5-FAM-TAACATCCCCGGGTCCGACTACATCAAT-BHQ-1. The primers and probes for RT-qPCR of β-Actin mRNA were as follows: ActB For, 5-AGCACAGAGCCTCGCCTTTGT-3; ActB Rev, 5-ATCATCATCCATGGTGAGCTGGC-3; ActB Pr, 5-HEX-ATCCGCCGC/ZEN/CCGTCCACAC- 3IABKFQ. Primers for RT qPCRs were designed to cover a splice junction and thereby only recognizing processed mRNA. The quantitation of RNaseP DNA was performed using 400 nM of each primer, 200 nM of probe and the Applied Biosystems TaqMan™ Universal PCR Master Mix (Thermo Fisher Scientific) in PCR program for RT was 50 °C for 3 min, denaturation at 95 °C for 10 min, and 45 cycles of 95 °C for 15 s and 60 °C for 60 s. The primers and probe for detection of RNaseP DNA have been described elsewhere [45]. The real-time PCR reactions were amplified on a Rotor-Gene 3000 thermal cycler (Corbett Research, Mortlake, Australia) and data were analyzed by Rotor-Gene software (v.6.1.93). Actin and RNase P were used as internal controls and data analysis was performed by ΔΔCT method [46].

### 2.5. Flow Cytometry

About 1 × 10^5^ cells were harvested for analysis of SHP-1 protein expression by flow cytometry. Cells were washed in PBS and fixed in 4% paraformaldehyde solution for 20 min at 4 °C using BD cytofix/cytoperm kit (BD Biosciences). Cells were washed twice in BD Perm/Wash buffer before staining with antibody. Cells were stained with FITC labeled goat anti-rabbit (IgG-FITC) antibody as staining control and PE-labeled SHP-1 antibody for 30 min in dark. Cells were washed twice in BD Perm/Wash buffer before running on the cytometer. FC analysis was performed using the BD^TM^ LSR II Fortessa flow cytometer system and the data was analyzed using the FACSDiva software. The relative amount of SHP-1 expression was determined by comparing the relative signal intensity of Jurkat and JHC.d cells.

### 2.6. Detection of Spliced SHP-1 mRNA

Total nucleic acid was extracted from the cells and contaminating genomic DNA was removed using DNase-treatment (RNeasy plus mini kit, Qiagen). cDNA was synthesized from an RNA template using the Superscript III First-Strand Synthesis Supermix (Invitrogen). Upstream primers specific to the SHP-1 transcript 1 and 3 were SHP-1-F, 5-CCGCACCTCGTCCAAACACA-3 and SHP-1L-F, 5- CCCGCACCTCGTCCAAGAGC-3, respectively. Downstream primer common to both transcripts was SHP-1-R; 5- TTAAATTACAAAAGAATGGGGCACTCCTA-3. For semi-quantitative PCR amplification, the following program was used: 94 °C for 4 min, 40 cycles of 95 °C for 30 s, 57 °C for 30 s 72 °C for 1 min and 72 °C for 5 min. Amplification products corresponding to SHP-1 transcript 1 (SHP-1, 239 bp) and 2 (SHP-1L, 383 bp) were detected on a 2% agarose gel.

### 2.7. Promoter CpG Island Identification and Primer Design

The identification of CpG island on SHP-1 P2 gene (NC_000012.12) was performed using Methyl Primer Express software v1.0 (Applied Biosystem). The CpG island with a GC percentage greater than 50% and with an observed /expected CpG ratio greater than 60% were selected. The DNA sequence of the CpG islands were modified by in silico bisulfite conversion and the primers against bisulfite template were designed using the same software.

### 2.8. Bisulfite Sequencing

The genomic DNA (gDNA) was extracted from Jurkat, C-expressing, THP1 and 293TT cells lines using QIAamp DNA mini kit (Qiagen). About 500 ng of purified gDNA was bisulfite treated with EZ DNA methylation-Gold kit^TM^ (Zymo Research Corporation, Täby, Sweden) according to the manufacturer’s instruction. The CpG islands on the SHP-1 P2 promoter were amplified with HotStarTaq DNA polymerase (Qiagen). The bisulfite treated DNA was used as template and amplified with bisulfite-specific forward and reverse primers respectively. For the CpG2 island, the following primer pair was used: SHP1_P2CpG2_For, ACACTCTTTCCCTACACGACGCTCTTCCGATCTNNNNNTAGTTTTTTGTTAGTTTTGGAGG and SHP1_P2CpG2_Rev, AGACGTGTGCTCTTCCGATCTAAAAAACAAATACACACTTATCCA. For the CpG1 island, the following primer pair was used: SHP1_P2CpG1_For, ACACTCTTTCCCTACACGACGCTCTTCCGATCTNNNNNGGGTTGTGGTGAGAAATTAATT and SHP1_P2CpG1_Rev, AGACGTGTGCTCTTCCGATCTCACACTCCAAACCCAAATAATA. For the proximal island, the following primer pair was used: Shp1P2P_For, ACACTCTTTCCCTACACGACGCTCTTCCGAlTCTNNNNNGTTTTTGAGTTTTTGATTGTAGA, and Shp1P2P_Rev, AGACGTGTGCTCTTCCGATCTTAAACCTCAAATACAACTCCC. For nested PCRs, the following primer pair was used: Nest_F, AATGATACGGCGACCACCGAGATCTACACGGAGTGGGACACTCTTTCCCTACACGACG, and Nest_R, CAAGCAGAAGACGGCATACGAGATTGGTGGTGGTGACTGGAGTTCAGACGTGTGCTCTTCCGATCT. The cycling conditions for the first PCR was as follows: 95 °C for 10 min, 35X (94 °C 30 s, 55 °C 30 s, and 72 °C 1 min) and 72 °C for 5 min. The second PCR was performed using following conditions: 95 °C for 10 min, 20 cycles of (94 °C 30 s, 55 °C 30 s, and 72 °C 1 min) and 72 °C for 5 min. The PCR products were treated with Affymetrix EXOSAP-IT enzyme (Thermo Fisher Scientific) and sequences were determined by the Sanger protocol (Eurofins Scientific, Konstanz, Germany). The approximate methylation level of cytosine residue at each CpG position was calculated by peak height measurement approach as described previously [47,48,49]. Determination of methylation status of specific genes by direct bisulfite-PCR sequencing compared to pyrosequencing and bisulfite-cloning sequencing is simple, high throughput and a reliable method [50]. For forward primer, methylation at each CpG site was calculated as 100 *C/(C + T) i.e 100 times the ratio between peak heights of C on the sequencing chromatogram and the sum of peak heights of C and T. Similarly, for reverse primer CpG methylation was calculated as 100 *G/(G + A) i.e 100 times the ratio between peak heights of G on the sequencing chromatogram and the sum of peak heights of G and A.

### 2.9. 5-Aza-2′-deoxycytidine (5-Aza) Treatment

Next, 5-Aza was dissolved in DMSO and a stock solution of 5 mM was prepared. Approximately 4 × 10^6^ Jurkat and the C protein-expressing cells were cultured and treated with the 5-Aza at a final concentration of 10 µM up to 5 days. Total RNA was extracted from the cells at 24, 48, 72 and 96 h post-treatment and mRNA expression was quantitated by RT-qPCR. Signals were adjusted for cellular DNA content by normalizing with the host marker RNase P as determined via qPCR.

### 2.10. Statistical Analysis

Data are expressed as mean values with standard deviation. Differences between multiple groups were performed by ANOVA and multiple comparisons tests. Differences between two groups were performed by multiple *t*-tests respectively. The significance level was set as *p* < 0.05. All tests were performed in Prism version 6 (GraphPad).

## 3. Results

### 3.1. Altered Tyrosine Phosphorylation of TCR-Related Signaling Molecules in the C Protein Expressing T Cells

**Figure 1:**
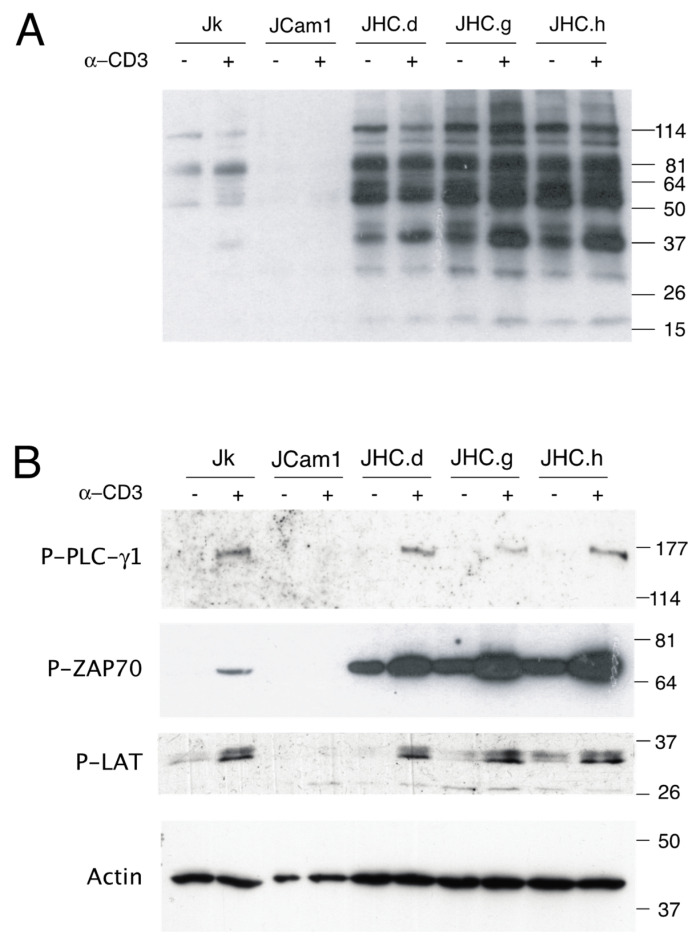
Altered tyrosine phosphorylation of the TCR-related signaling proteins in HCV C expressing cells. (**A**) Parental Jurkat (Jk), Lck kinase-deficient Jurkat derivative (J.Cam1), and C-expressing cells (JHC.d, JHC.g & JHC.h) were stimulated with an anti-CD3 antibody. Total tyrosine phosphorylation was examined by western blot using anti-phosphotyrosine (p-Tyr) antibody. (**B**) Specific tyrosine phosphorylation of the PLC-γ1, ZAP-70 and LAT proteins was detected with antibodies recognizing p-Tyr residues on respective protein by western blot. Actin serves as a loading control, the positions of molecular weight markers in (kDa) are indicated to the right.

Tyrosine phosphorylation is essential for proper T-cell signaling [51]. Since HCV infection modulates host cell immune response, we decided to investigate the effect of the HCV C protein on T-cell signaling [52]. To this end, we generated three individual C protein expressing Jurkat cell lines (JHC.d, JHC.g and JHC.h). As the tyrosine phosphorylation (p-Tyr) is an indicator of a T-cell signaling, we analyzed general p-Tyr signal in Jurkat, Lck kinase-deficient Jurkat derivative J.Cam1, and three individual C protein expressing cell lines (JHC.d, JHC.g and JHC.h) by western blotting. The J.Cam1 cells retain TCR expression, but are deficient in TCR signal transduction. Activation of the TCR with an anti-CD3 antibody resulted in a slight increase in general p-Tyr signal in Jurkat cells, whereas as expected no p-Tyr signal was detected in the kinase-deficient J.Cam1 cells (Figure 1A). In a striking contrast, all three C protein expressing cell lines showed high level of p-Tyr signal under non-activated conditions. TCR activation did not remarkably enhance p-Tyr signal, although some protein-specific p-Tyr signal was observed (Figure 1A, a protein close to 37 kDa marker).

The enhanced p-Tyr signal in the C protein expressing cells can be explained either by activation of the tyrosine kinase or by inhibition of a tyrosine phosphatase. Therefore, we examined p-Tyr status of the three signaling proteins: PLC-γ1, ZAP70 and LAT. A common feature between these proteins is that they are known targets for the tyrosine phosphatase SHP-1. While no basal phosphorylation of the SHP-1 targets was detected in Jurkat cells, triggering of the TCR with an anti-CD3 treatment resulted in p-Tyr signal detection in the PLC-γ1, ZAP70 and LAT proteins (Figure 1B). In contrast, no p-Tyr signal was detected in Lck-deficient J.Cam1 cells. Interestingly, the ZAP-70 and LAT proteins showed high basal level of p-Tyr signal in the C protein expressing cell lines. Furthermore, the p-Tyr signal was clearly increased in all three tested proteins after the TCR activation, although the extent of the p-Tyr signal varied between the individual cell clones (Figure 1B).

### 3.2. Specific Down-Regulation of the SHP-1 Protein Expression in the C Protein Expressing Cells

**Figure 2:**
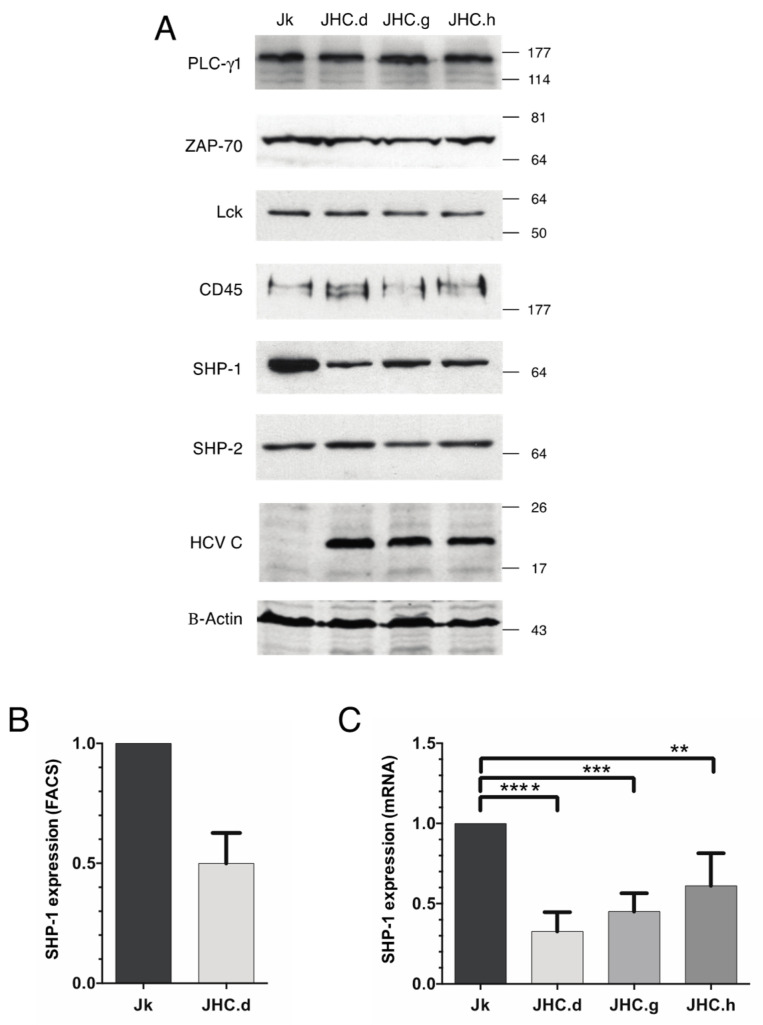
Specific downregulation of the SHP-1 protein and mRNA in the HCV C protein expressing cells. (**A**) Steady state levels of the PLC-γ1, ZAP-70, Lck, CD45, SHP-1, SHP-2 HCV C and actin proteins were analyzed in the parental Jurkat (Jk) and the C protein expressing (JHC.d, JHC.g & JHC.h) cells by western blot. (**B**) The SHP-1 protein expression in Jurkat (Jk) and JHC.d cells measured by flow cytometry. The experiment was performed twice and the data are presented as mean ± SD. (**C**) SHP-1 mRNA expression was analyzed by qRT-PCR from Jurkat (Jk) and three C expressing cells (JHC.d JHC.g & JHC.h). The relative expression levels of SHP-1 mRNA was normalized to the β-actin mRNA expression. The experiment was performed four times in duplicates and data are presented as mean ± SD. The statistical comparison of the expression levels was performed by one-way ANOVA and Bonferoni’s multiple comparison test. Bars with stars are statistically significant with adjusted *p* values ** *p* < 0.01, *** *p* < 0.001, **** *p* < 0.0001.

Accumulation of the p-Tyr signal in three SHP-1 target proteins (ZAP70, LAT and PLC-γ1) suggested us that the SHP-1 protein might be non-functional in the C protein expressing cells. To test it, we analyzed steady-state levels of the SHP-1 protein in the C protein expressing cell lines. Along with the SHP-1, expression of two other tyrosine phosphatases, CD45 and SHP-2 was analyzed. Remarkably, whereas the CD45 and SHP-2 expression were unaffected, the expression of the SHP-1 protein was selectively down-regulated in the C protein expressing cells (Figure 2A). As a comparison, no effect on steady-state levels of the tyrosine kinase targets ZAP70, Lck and PLC-γ1 was observed. Notably, similar reduction of the SHP-1 protein was observed when the SHP-1 protein levels were compared in the Jurkat and JHC.d cell lines by flow cytometry (Figure 2B). Since the reduced SHP-1 protein can be due to deficient mRNA synthesis, we quantitated SHP-1 mRNA levels in the parental Jurkat and C expressing cell lines by RT-qPCR. A significant decrease in SHP-1 mRNA level was detected in all individual C protein expressing cell lines compared to the parental Jurkat cells (Figure 2C). Taken together, our data indicate that constitutive expression of the C proteins specifically reduces accumulation of the SHP-1 mRNA in T cells.

### 3.3. SHP-1 mRNA Splicing Is Not Altered in the C Protein Expressing Cells

**Figure 3:**
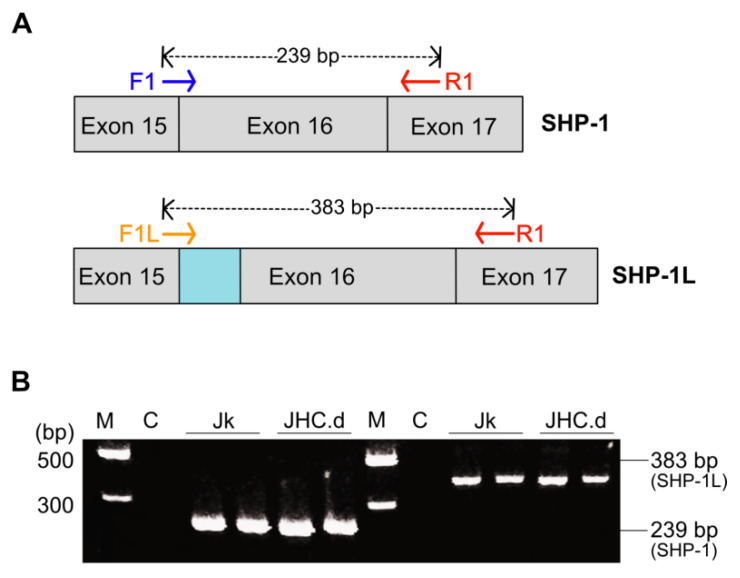
SHP-1 mRNA splicing is not altered in the HCV C protein expressing cells. (**A**) A schematic map showing the location of primers specific for SHP-1 (F1 and R1) and splice variant SHP-1 L (F1L and R1). The blue region at the splice junction between exon 15 and exon 16 corresponds to the unique coding sequence for SHP-1L due to the insertion of 144 nucleotide generated by alternative splicing. (**B**) Detection of SHP-1 and its splice variant SHP-1L in Jurkat (Jk) and C-expressing cells (JHC.d). PCR products; 239 bp amplicon (primers F1/R1) and 383 bp amplicon (primers F1L/R1) were detected on 2% agarose gel. Positions of molecular marker M in (bp) & C (negative control, no cDNA) are indicated in the right panel.

Analogously to many other human pre-mRNAs, the SHP-1 pre-mRNA undergoes alternative splicing [53]. In Jurkat cell, SHP-1 is the major mRNA transcript encoding a 68 kDa protein while the SHP-1L, generated by an alternative splicing event in exon 16 (Figure 3A), is a minor transcript encoding a 70 kDa protein [54]. The SHP-1L is 29 amino acid longer than SHP-1 and also lacks a potential tyrosine residue at Tyr-564 that could be important for the interaction with other signaling molecules [54,55]. To test whether the altered splicing pattern of SHP-1 pre-mRNA could be the reason for the distinct SHP-1 protein expression (Figure 2B), we designed primers to specifically detect respective SHP-1 splice variants. Both, SHP-1 and SHP-1L showed similar splicing pattern in the parental and the C protein expressing cells (Figure 3B), indicating that the C protein expression does not alter SHP-1 and SHP-1L mRNA splicing.

### 3.4. Reduced SHP-1 Expression Correlates with Its P2 Promoter Hypermethylation

The SHP-1 gene expression is often epigenetically controlled in T-cell lymphoma and leukemia through the promoter CpG methylation [35,37,39,40,41]. Also, previous studies have shown that the C protein is directly involved in epigenetic changes in HCC by altering CpG methylation pattern of tumor suppressor genes p16, E-cadherin and RASSFIA [56,57,58]. Hence, one possible explanation why the C protein expression reduced SHP-1 expression (Figure 2), is that the SHP-1 promoter CpG islands undergo specific, inhibitory CpG methylation modification [34]. To better understand the SHP-1 promoter methylation, we have examined three CpG islands (CpG2, CpG1 and proximal) on promoter 2 (P2) region of SHP-1 by bisulfite sequencing, which allows to identify methylated C nucleotides at a single nucleotide resolution. The reasons behind examining the aforementioned CpG islands is that they have high clusters of CpG motifs and methylation of the CpG dinucleotide motif in these islands has been previously reported in different types of hematological cancers and glioblastoma [35,36,39].

**Figure 4:**
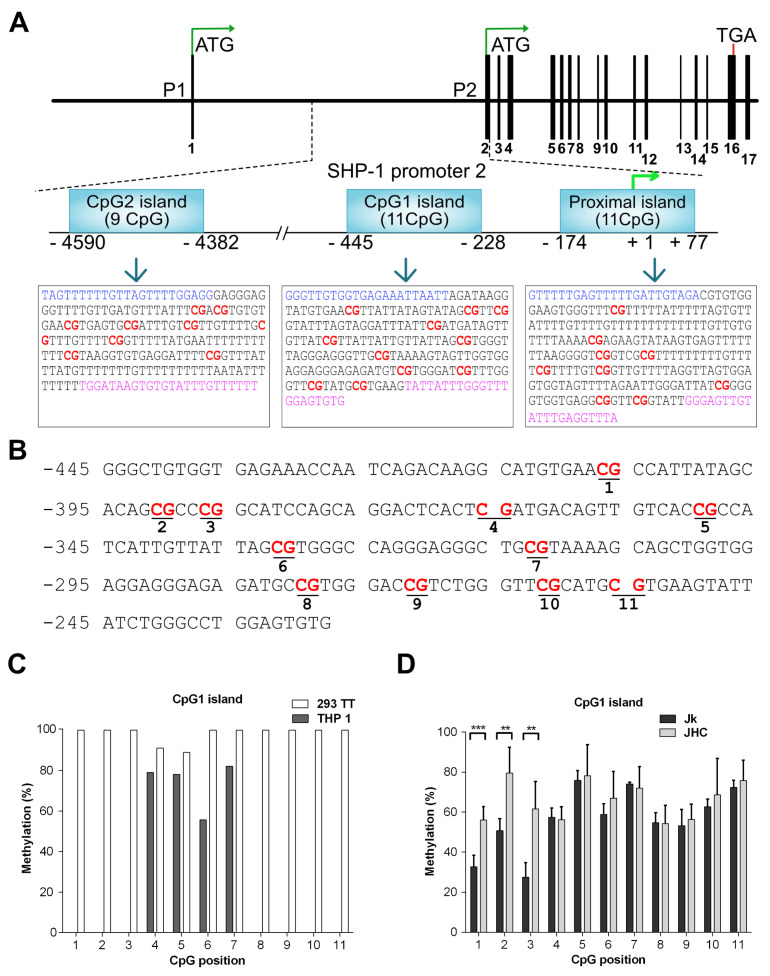
Alterations of SHP-1 P2 promoter methylation pattern in the C protein expressing cells. (**A**) Schematic map of SHP-1 gene and location of three CpG islands (CpG2, CpG1, proximal island) embedded into the SHP-1 promoter 2 (P2). The bisulfite converted sequences corresponding to each CpG islands are represented in the boxes. The CG dinucleotide (potential methylation site) is represented in red, whereas the blue and pink sequences represents the location of forward and reverse primers, respectively. (**B**) DNA sequence of the CpG1 island located between −445 to −228 relative to the SHP-1 transcription start site. CpG sites selected for methylation analysis are marked in bold and underlined. (**C**) Methylation at each CpG position within the CpG1 island as analyzed by bisulfite sequencing in hematopoietic THP-1 and non-hematopoietic 293TT cell lines. (**D**) Methylation analysis at each CpG position within the CpG1 island by bisulfite sequencing in Jurkat (Jk) and combined C expressing cell lines (JHC = JHC.d, JHC.g and JHC.h). Data are presented as mean ± SD of two sequencing runs performed in duplicates with both forward and reverse primers. For C expressing cells JHC (d,g,h) data are represented as sum of the averages of JHC.d, JHC.g and JHC.h clones. The statistical comparison of methylation levels between Jurkat and JHC cells were determined by multiple *t*-tests and the bars with stars were statistically significant with adjusted *p* values; ** *p* < 0.01, *** *p* < 0.001.

The proximal island (+77 to −174 bp) overlaps with the transcription start site of the gene and contains 11 CpG motifs, whereas the CpG1 island (−228 to −445 bp) lies upstream of the proximal island and also comprises 11 CpG motifs. (Figure 4A). In contrast, the CpG2 island (−4382 to −4590 bp) is positioned at the distal end of the SHP-1 gene and contains nine CpG motifs. The region of interest was amplified from the genomic DNA by using primers targeted against the bisulfite converted DNA in a two-step PCR reaction and Sanger sequenced (Figure 4B). To validate our experimental approach, we first analyzed CpG1 island methylation in two cell types: THP-1 (acute monocytic leukemia cell line, hematopoietic origin) and 293TT (human kidney epithelial, non-hematopoietic origin), where the tissue specific expression of SHP-1 is driven by two different and mutually exclusive promoters P2 and P1, respectively. The CpG1 island in 293TT is expected to be hypermethylated as the SHP-1 expression is directed from P1. The THP-1 cells can be considered as the a positive control for malignant cells of hematopoietic origin as the SHP-1 expression is regulated by P2 in these cells. Notably, inactivation of the P2 promoter by partial CpG1 methylation has previously been described in seveal transformed T cell lines [34]. As shown in Figure 4C, the THP-1 cells were unmethylated at CpG positions 1 to 3 and 8 to 11 and methylated at the CpG positions 4, 5, 6 & 7. In contrast, non-hematopoietic 293TT cells were fully methylated in all 11 CpG positions on CpG1 island (Figure 4C). To simplify the data visualization, methylation data from three individual C expressing cell lines (JHC.d, JHC.g and JHC.h) were combined into one data set (labelled as JHC). As shown in Figure 4D, the JHC cells showed clearly a different CpG methylation pattern. Although the methylation level at the CpG positions 4 to 11 was identical between the Jurkat and C-expressing cell lines, there was a significant difference in the CpG methylation at positions 1 to 3 in the CpG1 island. Notably, these particular CpG positions (1, 2, and 3) showed higher methylation in the C expressing cell lines (JHC) compared to the control, Jurkat cell line (Figure 4D).

**Figure 5:**
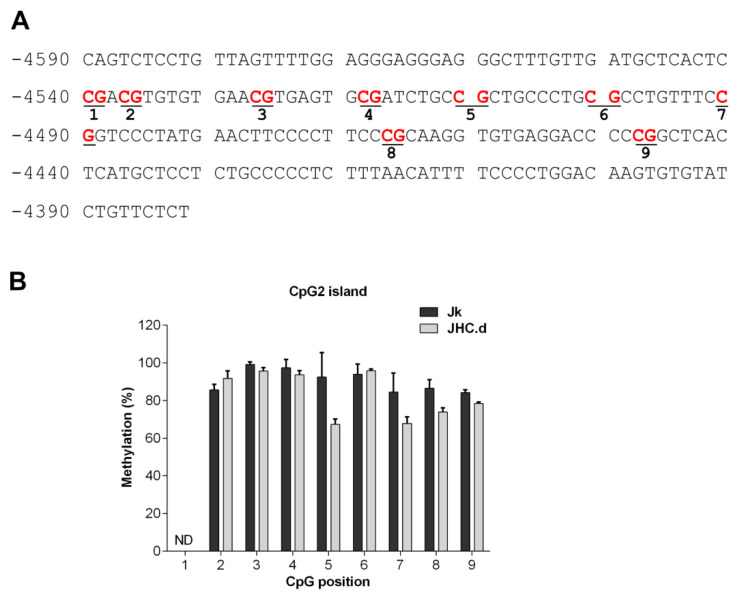
Methylation pattern in the CpG2 island of the SHP-1 P2 promoter. (**A**) DNA sequence of the CpG2 island located between −4590 to 4380 relative to the SHP-1 transcription start site. CpG sites selected for methylation analysis are marked in red and underlined. (**B**) Methylation at each CpG position within the CpG2 island as analyzed by bisulfite sequencing in Jurkat (Jk) and C expressing cell lines JHC.d. ND indicates not determined. Data are presented as mean ± SD of sequencing runs performed in duplicates with both forward and reverse primers. The statistical comparison of methylation levels between Jurkat and JHC.d cells were determined by multiple *t*-tests.

**Figure 6:**
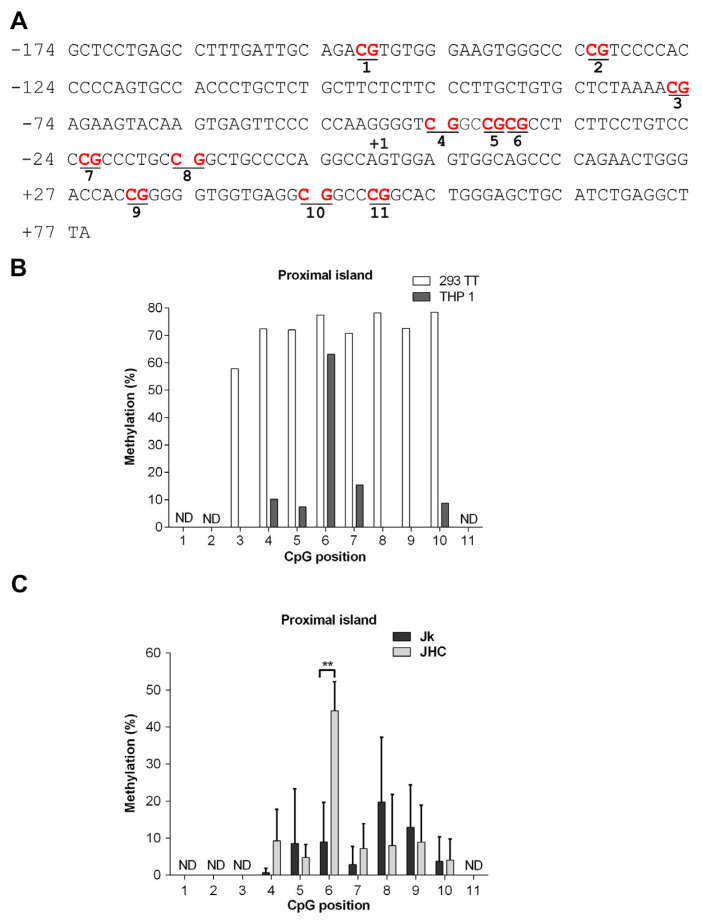
Methylation pattern in the proximal island of the SHP-1 P2 promoter. (**A**) DNA sequence of the proximal island located between −174 to +79 relative to the SHP-1 transcription start site. CpG sites selected for methylation analysis are marked in bold and underlined. (**B**) Methylation at each CpG position within the proximal island as analyzed by bisulfite sequencing in hematopoietic THP-1 and non-hematopoietic 293TT cell lines. (**C**) Methylation at each CpG position within proximal island as analyzed by bisulfite sequencing in Jurkat (Jk) and combined C expressing cell lines (JHC = JHC.d, JHC.g and JHC.h). ND indicates not determined. Data are presented as mean ± SD of two sequencing runs performed in duplicates with both forward and reverse primers. For C expressing cells JHC data are represented as sum of the averages of JHC.d, JHC.g and JHC.h clones. The statistical comparison of methylation levels between Jurkat and JHC (d,g,h) cells were determined by multiple *t*-tests and the bars with stars were statistically significant with adjusted *p* values; ** *p* < 0.01.

The CpG2 and proximal islands have been less extensively characterized regarding their CpG methylation pattern. For the CpG2 island, 8 of analyzed 8 CpG motifs were fully methylated in the parental Jurkat and JHC.d cells (Figure 5B). In contrast, various methylation patterns were observed for the proximal island (Figure 6A). In the 293TT cells, the proximal island was clearly hypermethylated at 8 of 8 analyzed CpG motifs (Figure 6B). Most of the CpG motifs in THP-1 cells displayed no (CpG positions 3, 8 and 9) or low (CpG positions 4, 5, 7 and 10) methylation, whereas the CpG position 6 was significantly hypermethylated in these cells (Figure 6B). Notably, significant hypermethylation of the CpG position 6 in the proximal island was also found in the JHC cells (Figure 6C). In contrast, this site was only marginally methylated in the parental Jurkat cells. For CpG position 4, 5 and 7 to 10 no significant CpG methylation difference was observed. For position 1 of the CpG2 island (Figure 5B) and positions 1 to 3 and 11 of the proximal island (Figure 6C) were we unable to obtain any interpretable data.

### 3.5. Responsiveness of the SHP-1 Gene Expression to 5-Aza-2’-deoxycytidine (5-Aza) Treatment

**Figure 7:**
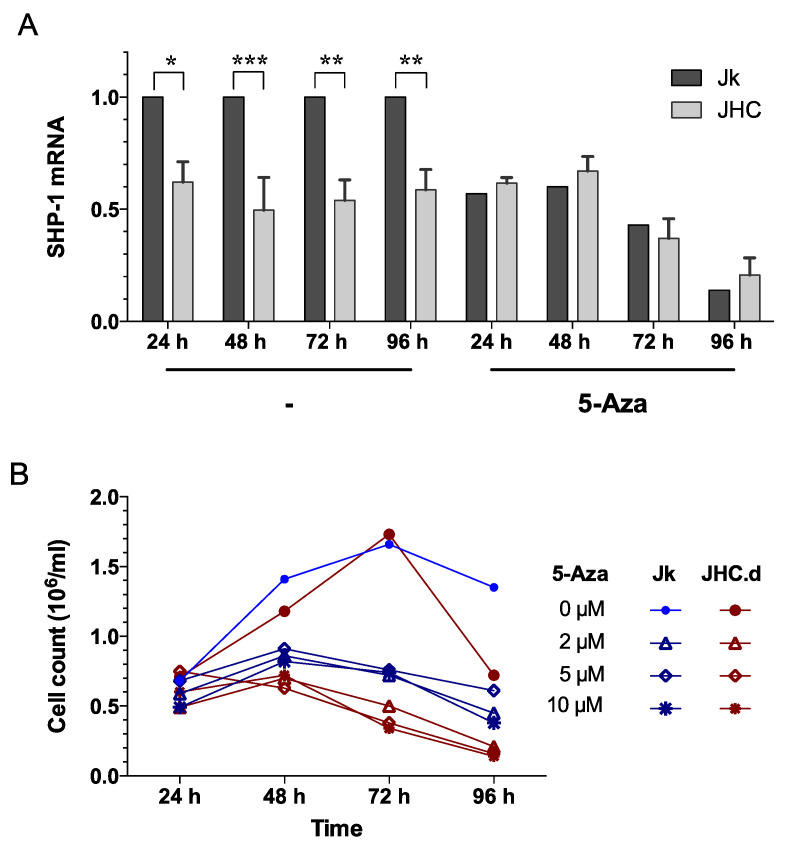
(**A**) Responsiveness of the SHP-1 gene expression to 5-Aza-2´-deoxycytidine (5-Aza) treatment. Jurkat (Jk) and C expressing cell lines (JHC = JHC.d, JHC.g and JHC.h) were treated with drug 5-Aza-2´-deoxycytidine (5-Aza) at 10 µM for 24, 48, 72 and 96 h. Total RNA was extracted from untreated and treated cells at each time point. SHP-1 mRNA expression was measured by qRT-PCR and normalized against the cellular gene RNase P. Data are presented as mean ± SD of triplicate experiments. Statistical significance was set as *p* < 0.05. Bars with stars were statistically significant as determined by two-way ANOVA multiple comparison test with Tukey’s correction. Adjusted *p* values; * *p* < 0.05, ** *p* < 0.01 and *** *p* < 0.001. (**B**) Cell proliferation of Jurkat (Jk, blue) and C-expressing cells (JHC.d, red). Cells were treated with different concentrations of 5-Aza and at indicated times cell proliferation was determined by trypan blue staining. Statistical comparison of the proliferation rate was performed by two-way ANOVA and Tukey´s multiple comparison test: 24 h, *p* = ns; 48 h, *p* = ns; 72 h *p* < 0.001; 96 h *p* < 0.01.

To determine if reduced expression of SHP-1 in the C protein expressing cells (Figure 2) is caused by the CpG methylation at the SHP-1 promoter, we treated cells with 5-Aza-2’-deoxycytidine (5-Aza), a known DNA methyltransferase inhibitor. Since 5-Aza blocks DNA methylation [59], we assumed that this treatment would have different effects on SHP-1 expression in the parental Jurkat and C expressing JHC cell lines. To this end, the expression of SHP-1 mRNA was measured in Jurkat and JHC (JHC.d, JHC.g and JHC,h) cells following 5-Aza treatment for 24, 48, 72 and 96 h. After treatment with 5-Aza, the expression of SHP-1 mRNA was equalized and no significant difference between the cell lines could then be observed (Figure 7A). Notably, whereas SHP-1 mRNA expression in the Jurkat cells was clearly down-regulated by the 5-Aza treatment, no such effect was observed in the C protein expressing cells at any tested time points (Figure 7A). The effect of 5-Aza treatment on cell proliferation was determined by trypan blue staining. As shown in Figure 7B, there was a clear difference between untreated and 5-Aza-treated cells, since the proliferative capacity was significantly decreased at two time points (72 and 96 h). Notably, the cytostatic effect of 5-Aza was more prominent in the JHC.d cells compare to the Jurkat cell, suggesting that cells expressing the C protein were more sensible to global CpG methylation changes. Collectively, our results indicate that the SHP-1 mRNA accumulation is regulated by DNA methylation and that the C protein expressing cells respond differently to 5-Aza treatment when compared to the parental cells.

## 4. Discussion

We have previously reported that stable expression of the HCV C protein caused increased basal levels of cytosolic [Ca^2+^] and altered TCR signaling in human T cells [17,18]. The present report extends the previous study by investigating more specifically the C protein effect on phosphotyrosine signaling and particularly on tyrosine phosphatase SHP-1. We found that basal and CD3-induced phosphorylation increased Tyr phosphorylation of the proteins involved in the proximal TCR signaling (Figure 1). A more detailed analysis with the p-Tyr specific antibodies showed that one particular protein, ZAP-70, was heavily hyperphosphorylated in our experimental system (Figure 1B). Based on the used site-specific p-Tyr antibodies, the Tyr residue 493 (Tyr493) was hyperphosphorylated in the C protein expressing cells. The ZAP-70 protein by itself is an essential tyrosine kinase in the TCR signaling and its activity is controlled by different phosphorylation events [60]. Phosphorylation of the Tyr493 residue is essential for the ZAP-70 protein function as mutation of this residue affected different TCR signaling steps. The non-phosphorylatable Tyr493 mutant is deficient in activating the nuclear factor of activated T lymphocyte (NFAT) signaling pathway in T cells [61]. In our previous study we showed that overexpression of the HCV C protein activates the NFAT signaling in T cells [18]. Interestingly, ZAP-70 hyperphosphorylation was more prominent in the C expressing cells compared to control, Jurkat, cells. Furthermore, ZAP-70 Tyr493 phosphorylation was also detected in the non-CD3-treated C protein expressing cells. Together, these observations suggest that expression of the C protein is a powerful way to induce Tyr493 phosphorylation, which in turn may have a positive effect on ZAP-70-dependent TCR signaling, including the NFAT signaling pathway in T cells.

Since a previous study has shown that ZAP-70 phosphorylation is regulated by the tyrosine phosphatase SHP-1 [62], we hypothesized that reduced activity of the SHP-1 protein might be responsible for the ZAP-70 hyperphosphorylation. Notably, our experimental data (Figure 2) confirmed this hypothesis. We found that enhanced p-Tyr signal in the ZAP-70 protein correlated with reduced expression of the SHP-1 protein (Figure 2A). Importantly, the C protein expression selectively downregulated only SHP-1 expression as the steady-state levels other tested tyrosine phosphatases (SHP-2, CD45) did not change. Our experiments also showed that reduced SHP-1 protein was due to the lower accumulation of the SHP-1 mRNA in the C protein expressing cells (Figure 2C). Reduced SHP-1 mRNA accumulation was not due to mis-splicing of the SHP-1 pre-mRNA as the two known splice variants, SHP-1 and SHP-1L [54,55,63], were equally well detected in the parental and the C protein expressing cells. Together, these data indicate that the C protein affected SHP-1 expression primary at the gene transcription level rather than targeting SHP-1 post-transcriptional modifications or the SHP-1 protein stability.

The SHP-1 gene expression is frequently suppressed or lost in diffuse large B-cell lymphoma (DLBCL), T-cell leukemias and lymphomas [35,64]. Previous studies have also shown that SHP-1 expression in DLBCL is controlled by DNA methylation and histone acetylation at the SHP-1 P2 promoter which comprises known binding sites for the transcription factors NF-κB [38], Sp1 [39], Oct-1 [39] and PU.1 [65]. Further, expression of the SHP-1 gene is suppressed in HTLV-1 transformed and tumor cells as a consequence of silencing of the SHP-1 P2 promoter by DNA methylation [35,38,39,66]. In line with these findings, we investigated if the methylation status of SHP-1 promoter can explain the reduced SHP-1 mRNA accumulation in the C protein expressing cells. Our bisulfite sequencing analysis of SHP-1 promoter P2 region revealed clear differences in the CpG methylation when the C protein expressing cells were compared to parental cells. First, in the CpG1 island, the first three CpG positions (1–3) showed a significant hypermethylation in the C expressing cells (Figure 4D). Second, in the proximal island, only the CpG position 6 was significantly hypermethylated in the C protein expressing cells (Figure 6C). Remarkably, this particular CpG position in the proximal island overlaps with the binding sequence for the transcription factor Sp-1 [39]. Based on these analyses there is a clear trend for specific CpG position methylation in the C protein expressing cells. Since the CpG methylation has a negative impact on gene transcription, we hypothesize that the C protein induces specific methylations in the CpG1 and proximal islands, which in concert with dislocation of Sp-1 from the promoter region affects SHP-1 gene transcription.

The CpG2 island was fully methylated in both the parental Jurkat and the C protein expressing cells (Figure 5). Previous studies have shown that SHP-1 CpG1 island is preferably hypermethylated in in high grade gliomas and malignant lymphomas/leukemias [36,67]. In contrast, in DLBCL, the CpG2 island has been shown to be mainly hypermethylated [35]. However, in these studies, methylation analysis at the individual CpG motifs was not shown. In this regard, our study is more specific as for the first time we show the quantitative differences at the individual CpG motifs in three CpG islands. Based on these data, we propose that the specific combinatorial hypermethylation at the CpG1 island (positions 1–3) and proximal island (position 6) significantly reduces SHP-1 gene expression in T cells.

**Figure 8:**
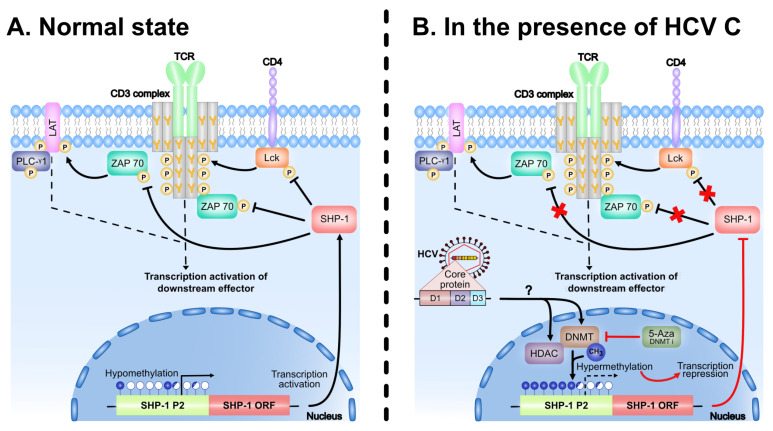
Feedback regulation of TCR signaling by the negative regulator SHP-1. (**A**) In the normal state, the stimulation of TCR by external stimuli results in the activation of PTKs (ZAP 70, Lck) and other signaling effectors (LAT, PLC-γ1) by phosphorylating the tyrosine residue (Y) on the protein molecules. SHP-1, which is constitutively associated with TCR complex, terminates the activation signal by removing the phosphate group from the protein molecule and maintains the TCR complex in a dephosphorylated state. The threshold set by the SHP-1 together with PTKs is therefore critical for the proper signaling of downstream targets. (**B**) The HCV C protein disturbs the homeostasis by downregulating the expression of SHP-1 via CpG methylation of its promoter. C protein inactivates the SHP-1 expression, either by inducing the DNMTs and, thereby methylating the SHP-1 promoter, or/and by HDAC-mediated chromatin modifications. The CpG methylation can be blocked by the drug 5-Aza. The CpG islands and potential methylation sites are represented by the lollipops (in blue). The open, filled and half filled lollipops represents the unmethylated, fully methylated and partial methylated CpG’s, respectively. Inhibition of the SHP-1 phosphatase activity of is represented by the crosses (in red). TCR, T cell receptor; PTK, protein tyrosine kinases; DNMTs, DNA methyl transferases; HDAC, histone deacetylases.

Our present model how SHP-1 activity is regulated by the HCV C protein is summarized in Figure 8. In line with the proposed model, the multifunctional HCV C protein has been reported to induce hypermethylation of promoters of different tumor suppressor genes involved in HCC via modulation of DNMTs and histone deacetylases [56,57,58,68,69,70]. Interestingly, elimination of CpG methylation by 5-Aza treatment did not enhance SHP-1 expression in our experimental system (Figure 7A). This observation may indicate that in addition to the CpG methylation also specific histone modifications, such as histone deacetylation, may block SHP-1 expression in the C protein expressing cells. Alternatively, the net effect after treatment with 5-Aza could be a consequence of general toxicity that reduces SHP-1 gene expression in the C protein expressing cell lines (Figure 7B). Hence, the future studies with combinatorial usage of 5-Aza and different histone modifying drugs should reveal more precisely the mechanism(s) how the C protein blocks SHP-1 gene expression by targeting the P2 promoter.

## 5. Conclusions

In conclusion, our results demonstrate that expression of the HCV C protein induces a very specific CpG methylation pattern at the SHP-1 P2 promoter resulting in reduced SHP-1 gene expression and altered phosphotyrosine signaling including the ZAP-70 protein. Since the biological relevance of our findings in the context of HCV infection remains to be established, further studies are warranted and may contribute to the understanding of HCV pathogenesis.

## Data Availability

Not applicable.
